# Adolescents with same-sex interest: experiences of sexual harassment are more common among boys

**DOI:** 10.1080/21642850.2019.1598864

**Published:** 2019-03-28

**Authors:** Riittakerttu Kaltiala-Heino, Nina Lindberg, Sari Fröjd, Henna Haravuori, Mauri Marttunen

**Affiliations:** a Department of Adolescent Psychiatry, Tampere University Hospital, Tampere, Finland; b Vanha Vaasa Hospital, Vaasa, Finland; c Faculty of Medicine and Health Technology, Tampere University, Finland; d Forensic Psychiatry, Helsinki University and Helsinki University Hospital, Helsinki, Finland; e Faculty of Social Sciences, Tampere University, Finland; f Department of Adolescent Psychiatry, Helsinki University Hospital, Helsinki, Finland; g Adolescent Psychiatry, Helsinki University and Helsinki University Hospital, Helsinki, Finland

**Keywords:** Sexual harassment, GLBT, adolescence

## Abstract

**Purpose:** To explore whether sexual harassment experiences are more common among adolescents reporting romantic and erotic interests in the same sex and both sexes, when sociodemographic and mental health confounding are controlled for, and whether the associations are similar in both sexes and in different phases of adolescence.

**Methods:** A cross-sectional survey among a nationally representative dataset of 25,147 boys and 25,257 girls in comprehensive school, and 33,231 boys and 36,765 girls in upper secondary education. Self-reports of experiences of sexual harassment, and emotional (depression) and behavioral (delinquency) symptoms were used.

**Results:** All associations between sexual minority status and harassment diminished clearly when mental disorder dimensions were controlled for. In the comprehensive school sample (mean age 15.4 years), sexual harassment experiences were 4–7-fold more common among boys, and 1.5–3-fold among girls, with same-sex/both-sexes interest, compared to those interested exclusively in the opposite sex. In the upper secondary education sample (mean age 17.4 years), among boys, sexual harassment was reported 3–6-fold more commonly by those not exclusively heterosexually interested. Among older girls, a slight increase in sexual harassment experiences was seen among those interested in both sexes.

**Conclusions:** Sexual harassment experiences are associated with sexual minority status, particularly among boys. Confounding by mental disorders needs to be accounted for when studying sexual minority status and sexual harassment.

## Introduction

The legal definition of sexual harassment is gender-based discrimination which creates a hostile work/school environment and may seriously impair the victim’s performance at work or ability to participate in and benefit from education (Fineran, 2002; Gruber & Fineran, 2007; Pina, Gannon, & Saunders, 2009). Sexual harassment can be divided into gender harassment, unwelcome sexual attention and sexual coercion (Buchanan, Bluestein, Nappa, Woods, & Depatie, 2013; Fitzgerald, Gelfand, & Drasgow, 1995). Gender harassment entails verbal and non-verbal gender–based hostile/derogatory communication or gender related name-calling. Unwelcome sexual attention includes any sexual behavior, propositions, invitations etc. which are distasteful and unwelcome to the target and perceived as offensive. Sexual coercion includes actual sexual assault but also any behavior attempting to extort sexual compliance by means of promises/benefits or threats.

Subjection to sexual harassment is not uncommon during the years of adolescent development. Between one to two thirds of adolescents in various school and college samples report being subjected to sexual harassment (Chiodo, Wolfe, Crooks, Hughes, & Jaffe, 2009; Gruber & Fineran, 2007; Kaltiala-Heino, Frojd, & Marttunen, 2016b; Petersen & Hyde, 2009). Subjection to sexual harassment is associated with a variety of negative psychosocial outcomes and mental disorders (American Association of University Women, 2001; Bucchianeri, Eisenberg, Wall, Piran, & Neumark-Sztainer, 2014; Kaltiala-Heino, Frojd, & Marttunen, 2016a), and appears to be more detrimental to adolescent mental health than other types of harassment (Bucchianeri et al., 2014). Thus, subjection to sexual harassment in adolescence is a significant problem, and in order to prevent it and to reduce harm caused by it, more understanding is needed about the factors that predispose adolescents to becoming subjected to sexual harassment. Sexuality and sexual behavior appear to be among such factors (Kaltiala-Heino, Savioja, Fröjd, & Marttunen, 2018).

Sexual development accelerates in adolescence. Young people’s experiences of a changing body, sexuality, and gender identity affect intrapersonal, relational, and societal interactions (Romeo & Kelley, 2009). Romantic and erotic interests and behavior mature gradually towards adulthood (Madkour, Farhat, Halpern, Godeau, & Gabhainn, 2010; Kotchick, Shaffer, Forehand, & Miller, 2001; Savioja, Helminen, Fröjd, Marttunen, & Kaltiala-Heino, 2015). An important facet of sexual maturation is sexual orientation. Sexual orientation comprises the aspects of attraction, behavior, and identity (Adelson & the American Academy of Child and Adolescent Psychiatry (AACAP) Committee on Quality Issues (CQI), 2012; Saewyc et al., 2004). A majority of adolescents and adults are attracted to and have romantic and erotic relations with members of the opposite sex and identify as heterosexuals (Centers for Disease Control and Prevention, 2016; Smith, Rissel, Richters, Grulich, & de Visser, 2003). Sexual minority youth refers to adolescents with not exclusively heterosexual sexual orientation. Puberty itself, romantic and erotic interests intensifying in puberty, and advancing sexual experiences during adolescent development are stressful and associated with increased prevalence of emotional and behavioral disorders (Kaltiala-Heino, Kosunen, & Rimpela, 2003; Kaltiala-Heino, Marttunen, & Fröjd, 2015; Savioja et al., 2015), but it is likely still more stressful to become aware of same-sex erotic and romantic interests, or of bisexual orientation, and to seek non-heterosexual romantic and erotic contact. This additional stress may be related to heterosexism.

Heterosexism refers to a systematic process of privileging heterosexuality relative to other sexualities, based on the assumption that heterosexuality and heterosexual power and privilege are normal and ideal (Chesir-Teran, 2003; Chesir-Teran & Hughes, 2009; Szymanski, Kashubeck-West, & Meyer, 2008). This includes explicitly expressed prejudiced attitudes and ideologies but also contextual processes that privilege one group over others, for example by reinforcing invisibility of sexual minorities or not intervening in expressions of discrimination against or victimization of sexual minority individuals or groups (Chesir-Teran, 2003; Chesir-Teran & Hughes, 2009). Heterosexism has been suggested to be an important reason for subjection to discrimination and abuse experienced by sexual minority people (Katz-Wise & Hyde, 2012). Sexual harassment has been suggested to arise from and reinforce heterosexism (Pina et al., 2009; Street, Gradus, Stafford, & Kelly, 2007). Adolescents commonly use verbal expressions that qualify as gender harassment, referring in a pejorative manner to sexuality and same-sex interest. This serves both to perpetrate aggression and to gain social dominance among friends (Poteat & DiGiovanni, 2010; Poteat & Rivers, 2010). Regardless of the target’s actual or assumed sexual orientation, such language suggests a negative attitude towards homosexuality in general, and further emphasizes the strength of the heterosexual norm (Chesir-Teran & Hughes, 2009; Poteat & DiGiovanni, 2010; Poteat & Rivers, 2010). Despite the contemporary advances in the rights and visibility of sexual minorities, heterosexism is widespread (Dunn & Szymanski, 2018) and this implies that adolescents developing towards a non-heterosexual orientation are likely to be more vulnerable to becoming targets of sexual harassment than their heterosexually developing peers.

Some studies have indeed reported that sexual minority adolescents experience more sexual harassment than their heterosexual peers (Gruber & Fineran, 2008; Mitchell, Ybarra, & Korchmaros, 2014; Williams, Connolly, Pepler, & Craig, 2003). Sexual minority youth have also been found to report disproportionately common experiences bullying (Burton, Marshal, Chisolm, Sucato, & Friedman, 2013; Friedman et al., 2011; Katz-Wise & Hyde, 2012) of which a considerable share is of sexual nature (Ashbaug & Cornell, 2008). This may relate to heterosexist (homophobic) discrimination of a non-heterosexual adolescent’s self-expression and serve to strengthen heteronormativity (Chesir-Teran & Hughes, 2009). However, studies focusing specifically on associations between sexual orientation and subjection to sexual harassment are to the best of our knowledge few, this issue has only been studied in North American samples, and mainly in samples of only several hundred participants, including rather small numbers of non-heterosexual young people (Gruber & Fineran, 2008; Mitchell et al., 2014; Williams et al., 2003, studying respectively samples recruited from New England, in the Internet, and in Toronto, Kingston & Montreal). More research is needed on associations between sexual orientation and subjection to sexual harassment among adolescents, in large non-selected samples and also from outside of North America.

Sexual minority youth display an excess of both emotional and behavioral disorders (Beaver et al., 2016; Birkett, Espelage, & Koenig, 2009; Marshal et al., 2011). According to minority stress theory (Meyer, 1995, 2013), sexual minorities experience excessive stress not only due to personal subjection to discrimination and victimization, but also due to internalized heterosexism (internalized homophobia) and perceived stigma. Internalized heterosexism refers to having internalized the mainstream negative attitudes and beliefs about sexual minorities, which results in self-labeling. Perceived stigma again results in anticipating and being vigilant for personal discrimination and victimization. Internalized homophobia and excessive vigilance for discrimination create a stressful social environment and chronic stress, which increases the risk of mental health problems (Meyer, 1995, 2013). Adolescents are particularly sensitive to how they meet societal expectations (Rotheram-Borus & Fernandez, 1995). Emerging sexuality and societal expectations regarding sexual orientation may cause developmental challenges for sexual minority adolescents (Adelson & the American Academy of Child and Adolescent Psychiatry (AACAP) Committee on Quality Issues (CQI) 2012; Rotheram-Borus & Fernandez, 1995). This may increase the risk of emotional and behavioral problems even without actual external discriminatory events, and particular sensitivity to clues suggesting discrimination and rejection, such as sexually harassing interactions.

To summarize, experiences of sexual harassment (Kaltiala-Heino et al., 2016a) and of belonging to sexual minorities are associated with both emotional (Marshal et al., 2011) and also behavioral (Beaver et al., 2016) disorders. Emotional and behavioral disorders may increase hostile attribution bias, proneness to perceive negative communications even in neutral interactions, and sexual minority youth may be prone to attribution bias also due to minority stress. Emotional and behavioral symptom dimensions should be controlled for in order to explore independent associations (main effects) between belonging to a sexual minority and experiences of sexual harassment. Experiences of sexual harassment and mental disorders are also both associated with sociodemographic adversities (Kaltiala-Heino et al., 2016b), and therefore confounding by sociodemographics should be controlled for when exploring the main effects of potential correlates on sexual harassment.

Self-expression deviating from the masculine norm among boys is less readily tolerated than deviating from the feminine norm among girls (Ristori & Steensma, 2016). Experiences of sexual harassment are less common but more strongly associated with mental disorders in boys than in girls (Kaltiala-Heino et al., 2016a, 2016b). Heterosexism and sexual harassment serve to maintain not only superiority of heterosexuality over other sexual orientations but also the dominant position of heterosexual males (Pina et al., 2009; Street et al., 2007; Szymanski et al., 2008). Minority stress may consequently also have a greater impact of non-heterosexual boys’ perception of social interactions than that of sexual minority girls. Given these, gender differences can be expected in the associations between sexual minority status and sexual harassment. Finally, sexuality, behavioral controls, and abilities in perspective taking all develop throughout adolescence (Moshman, 2011; Cacciatore, Korteniemi-Poikelainen, & Kaltiala-Heino, 2018 (under review); Steinberg, 2005). Adolescents of different ages likely perceive social interactions differently, and therefore the associations between sexual minority status and conceptions of experiencing sexual harrassment may differ between adolescents of different ages. It has, for example, earlier been shown that involvement in school bullying decreased with age both among heterosexual and sexual minority youths (Kurki-Kangas, Marttunen, Fröjd, & Kaltiala-Heino, 2018). Similar development could be expected regarding sexual harassment.

The aim of our study was to explore the associations between non-heterosexual romantic and erotic attraction and experiences of sexual harassment in a large, nationally representative data of 14–18-year-old adolescents in Finland. We hypothesized that

1. Romantic/erotic interests other than exclusively heterosexual are associated with increased experiences of sexual harassment, and that this will be seen across different types of sexual harassment.
2. The associations between same-sex and both-sex romantic/erotic interests and sexual harassment experiences are stronger among boys than girls.
3. The associations between not exclusively heterosexual romantic/erotic interests and experiences of sexual harassment will be stronger among younger adolescents who have weaker behavioral controls and less developed coping skills.
4. The associations detected at the bivariate level will grow considerably weaker when mental disorders and socio-demographics are controlled for, indicating that the associations between not exclusively heterosexual romantic/erotic interests and experiences of sexual harassment are partially mediated by these confounders.

## Materials and methods

The School Health Promotion Study (SHPS) of the National Institute for Health and Welfare is a school-based survey designed to examine the health, health behaviors, and school experiences of teenagers. The survey is sent to every municipality in Finland, and the municipalities decide if the schools in their area will participate in the survey. The survey is run primarily for health policy and administrative purposes, and the data is available for scientific research on request. The main aim of the survey is to produce national adolescent health indicators that municipalities can utilize in planning services and that can be used at national level to assess the effectiveness of health policies. The authors obtained permission to use data for scientific research but have not been responsible for collecting it.

The survey is conducted among 8th and 9th graders of comprehensive school and 2nd year students of upper secondary education (upper secondary school and vocational school). In 2015, the SHPS data comprised 50,404 responses from comprehensive schools, 38,760 from upper secondary schools and 31,236 from vocational schools. This covers 64% of all 8th and 9th graders and 43% of all upper secondary education (upper secondary school and vocational school) students in Finland (Halme, Kivimäki, Luopa, & Matikka, 2016). In an analysis of the sampling made by National Institute for Health and Welfare (Halme, Kivimäki, Luopa, & Matikka, 2016), the data were evaluated to be of high quality and representative of the Finnish adolescent population. Participants completed the questionnaire anonymously online during a school lesson under the supervision of a teacher, who did not interfere with the responses. Participants were informed in both oral and written form about the nature of the study as well as the voluntary nature of participation, and further that returning the survey would be taken to be consent to participate. The questionnaires took 30–45 min to complete. The study had been duly approved by the ethics committee of Pirkanmaa Hospital District and the National Institute of Health and Welfare. The respondents were advised to talk with their parents or contact school health and welfare services (school nurse, doctor, psychologist or social worker), available in all schools in Finland if they wished to discuss further anything elicited in the survey. The respondents in the comprehensive school sample were 25,147 boys, mean (sd) age 15.4 (0.66) years and 25,257 girls, mean (sd) age 15.3 (0.64) years. The respondents in the upper secondary education group were 33,231 boys, mean (sd) age 17.4(0.74) years and 36,765 girls, mean (sd) age 17.5 (0.81) years.

### Measures

#### Romantic and erotic interests

Adolescents in the 8th and 9th grades of comprehensive school were asked ‘Have you had a crush on or been in love with … ’, with response alternatives yes, girl(s)/yes, boy(s)/yes, both girl(s) and boy(s)/no I haven’t/ don’t know. Those in upper secondary school/vocational school were asked ‘Are you sexually interested in … ’ with response alternatives females/males/both females and males/neither females nor males /I don’t know.

#### Sexual harassment

The adolescents were asked if they had experienced any of the following: (1) Disturbing sexual propositions or harassment at school, hobbies, on the street, in shopping malls or other public spaces; (2) Disturbing sexual propositions or harassment via telephone or the Internet; (3) Bullying, name-calling or criticism that insulted their body or sexuality (4) Being touched in intimate body parts against their will; (5) Being pressured or coerced into intercourse or other sexual activity; (6) Being offered money, goods or drugs/alcohol in return for sex. The response options to each question were: yes, repeatedly/yes, sometimes/no. The six items were all statistically significantly inter-correlated at level *p* < 0.01, Pearson’s correlation coefficients ranging from 0.49 (between bullying or sexual name-calling and being offered payment for sex) to 0.76 (between being touched in intimate body parts and being coerced or pressured to engage in sex), and when explored as a scale, the six items displayed good internal consistency (Cronbach alpha 0.88). The six items were classified to gender harassment, unwelcome sexual attention and sexual coercion (Buchanan et al., 2013; Fitzgerald et al., 1995). Gender harassment was recorded if the respondent answered that s/he had repeatedly experienced sexual name-calling (question 3). Unwelcome sexual attention was recorded if the respondent reported that s/he had repeatedly experienced disturbing sexual propositions (questions 1 and/or 2) or being touched in intimate body parts against his or her will (question 4). Sexual coercion was recorded if the respondent reported that s/he had repeatedly been pressured or coerced into sex or offered payment for sex (questions 5 and 6).

### Emotional and behavioral symptoms

The emotional and behavioral symptoms elicited were depression, generalized anxiety, and delinquent behavior.

Depression was measured with two screening questions focusing on the two main criteria of major depression: (1) ‘During the past month, have you often been bothered by feeling down, depressed, or hopeless?’ and (2) ‘During the past month, have you often been bothered by little interest or pleasure in doing things?’ Both had response alternatives yes (coded 1) and no (coded 0). These two questions have shown good psychometric properties in detecting depression in primary care in adults and adolescents (Richardson et al., 2010; Whooley, Avins, Miranda, & Browner, 1997).

Generalized anxiety symptoms were elicited by GAD-7, a self-report questionnaire designed to identify probable cases of generalized anxiety disorder and to assess symptom severity. The GAD-7 items describe the most prominent diagnostic features of the DSM IV diagnostic criteria for generalized anxiety disorder. The GAD-7 elicits how often, during the last two weeks, the respondent has been bothered by each of the seven core symptoms of generalized anxiety disorder. Response options are ‘not at all,’ ‘several days,’ ‘more than half the days,’ and ‘nearly every day,’ scored respectively as 0, 1, 2, and 3. The GAD-7 has been shown to be a reliable and valid measure for detecting generalized anxiety disorder in primary care and population (Kujanpaa et al., 2014; Lowe et al., 2008).

Delinquent behavior was elicited with six questions:

During the past 12 months have you (1) drawn tags or graffiti on walls or elsewhere, (2) deliberately damaged or destroyed school property or the school building, (3) deliberately damaged or destroyed other property, (4) stolen from a shop or a stall, (5) been involved in a fight, (6) beaten someone up?

All these had response options no (=0)/once (=1)/2–4 times (=2)/more than 4 times (=3) and a sum score was formed of the delinquent behaviors, theoretically ranging from 0 to 24. The self-report questions on delinquent behavior were adopted from the Finnish Self-Report Delinquency Study questionnaire, which is a modiﬁed version of the International Self-Report Delinquency Study (ISRD) instrument (Junger-Tas, Terlouw, & Klein, 1994). The ISRD instrument has been shown to possess adequate reliability in test–retest studies (Zhang, Benson, & Deng, 2000).

### Invalid responses

It has been demonstrated that adolescents exaggerate their belonging to minorities or having negative experiences, and that bias due to such invalid responding can be reduced by excluding respondents reporting implausible combinations of extreme responses beyond the focus of interest for the current analyses in topics theoretically not related to variables of interest for the actual study questions (Robinson-Cimpian, 2014). We accordingly excluded from the final analyses respondents who had reported suffering at school from six out of eight negative physical conditions of the built environment (overcrowding, noise, poor light, poor air quality, inappropriate temperature, dirt, poor chairs, uncomfortable restrooms) and reportedly brushing their teeth less than weekly. The screening variable so created was in both educational groups and both sexes associated with reporting same-sex romantic and erotic interest at a statistical significance level of *p* < 0.001. Using this criterion resulted in the exclusion of 1.6% of the boys and 0.4% of the girls in the comprehensive school sample, and of 0.6% of the boys and 0.1% of the girls in the upper secondary school or vocational school sample.

### Covariates

Sociodemographic variables used were family structure, mother’s and father’s education, and unemployment in the family. These covariates were used because it has previously been shown that they are associated with experiences of sexual harassment in adolescent population (Kaltiala-Heino et al., 2016, 2016b), and they are also correlates of emotional and behavioral disorders (Hill, 2002; Torikka et al., 2014).

The adolescents were asked if they lived with their mother and father together/with mother and father alternately/with mother alone/with father alone/with mother or father and her/his partner/in a foster family/with some other guardian/in a child welfare institution/with some other adult or adults/some other arrangement (clarification requested). In the analyses family structure was dichotomized to mother and father together/any other family constellation. Of the adolescents, 63.8% were living with both parents.

Parental education was elicited separately for the father and the mother: ‘What is the highest educational qualification your father/mother has completed?’ The response alternatives were comprehensive school only/upper secondary school or vocational school/upper secondary school or vocational school and further vocational studies/university or university of applied sciences. The parental educational level was coded low if the parent had completed only comprehensive school. Of the adolescents, 6.7% reported low education of mother, and 11.0% low education of father.

Parental unemployment was elicited with the question ‘During the past year, have your parents been unemployed or laid off work?’ The response alternatives were no/one of the parents/both parents. Of the respondents, 67.3% reported no parental unemployment during the past year, 28.8% reported that one parent had been unemployed or laid off, and 3.9% reported that both parents had been unemployed or laid off during the past year.

### Statistical analyses

The distribution of romantic and erotic interests is given in Table 1 and that of reported experiences of sexual harassment in Table 2. Associations between romantic and erotic interests and experiences of sexual harassment were first studied using cross-tabulations with chi-square statistics. Logistic regression was used to study multivariate associations. The romantic/erotic interest variable was used as the independent variable, and opposite-sex interest was used as the reference category. Gender harassment, unwelcome sexual attention, and sexual coercion were entered in turn as the dependent variable. As covariates, first age (continuous), and then age, depression, anxiety, and delinquency were entered. Finally, sociodemographic variables were controlled for. Odds ratios (OR) with 95% confidence intervals (95% CI) are given. All the analyses were run separately for boys and girls, and for comprehensive school and upper secondary education groups. Due to the large size of the data, and in order to avoid bias related to multiple testing, we set the limit for statistical significance at *p* < 0.001.

**Table 1.** Romantic and erotic interests among 8th and 9th grade pupils of comprehensive school and among 2nd year students in upper secondary/vocational school in Finland (%).

Has had a crush on or been in love with	Opposite sex	Same sex	Both sexes	None	Doesn’t know	Missing
Boys 8th and 9th grade (*n* = 24738)	74.3	1.6	1.8	11.9	10.2	0.7
Girls 8th and 9th grade (*n* = 25147)	75.4	1.5	6.4	9.0	7.7	0.4
Is sexually interested in	opposite sex	same sex	both sexes	none	doesn’t know	missing
Boys 2nd year upper secondary/vocational school (*n* = 33040)	94.1	1.3	2.1	0.8	1.6	0.2
Girls 2nd year upper secondary/vocational school (*n* = 36732)	87.4	2.0	6.8	0.9	2.8	0.1

**Table 2.** Distribution of experiences of repeated gender harassment, unwelcome attention and sexual coercion (% (*n*/*N*)).

	Boys 8th and 9th grade	Girls 8th and 9th grade	*p* boys vs. girls	Boys 2nd year upper secondary/vocational school	Girls 2nd year upper secondary/vocational school	*p* boys vs. girls
Gender harassment	3.5 (845/24384)	3.1 (783/24954)	<0.001	2.5 (833/32684)	2.6 (940/36553)	<0.001
Unwelcome sexual attention	3.6 (895/24738)	4.1 (1020/25147)	0.006	3.1 (1014/330404)	4.1 (1490/367832)	<0.001
Sexual coercion	2.7 (670/24783)	1.6 (392/25147)	<0.001	2.2 (720/33040)	1.4 (531/36723)	<0.001

### Attrition

Of boys (girls) in the comprehensive school sample, 0.7% (0.4%) had not responded to the question eliciting romantic interest (*p* < 0.001). Not responding to this item was not related to sociodemographic or mental health variables. Of boys (girls) in upper secondary education, 0.2% (0.1%) (*p* = ns) had not responded to the question eliciting sexual orientation. Not responding on this was statistically significantly (*p* < 0.001) associated with not living with both parents, but in practice the difference was negligible (0.2% vs. 0.1%). Otherwise, no statistically significant associations were detected with sociodemographic or mental health variables.

Of the respondents, 581 (0.5%) had left unanswered all the questions concerning sexual harassment. Boys had omitted this section more frequently than girls (0.7% vs. 0.3%, *p* < 0.0001). Not responding to sexual harassment items was statistically significantly (*p* < 0.001) associated with mother’s low level of education, both parents having been unemployed or laid off, and not living with both parents, but in practice the differences between groups on these sociodemographic variables were 0.1–0.3% and thus negligible. Those ignoring all sexual harassment items scored higher on delinquency than those responding (mean (sd) 9.5 (5.1) vs. 7.7 (2.3), *p* < 0.001).

## Results

### Romantic/erotic interests and experiences of sexual harassment (Hypothesis 1)

The proportion of those reporting recurring sexual harassment experiences varied from 1.4% (sexual coercion among girls in upper secondary education) to 4.1% (unwelcome sexual attention among girls of both age groups) (Table 2).

In the comprehensive school sample among both sexes, all types of sexual harassment experiences were more common among those reporting interest in the same sex or both sexes than among those exclusively heterosexually interested, those unsure if they had been in love with or had a crush on someone or those who had not been in love, with greater differences between groups among boys. In the upper secondary education sample, among boys all forms of sexual harassment were more commonly reported by those interested in the same sex, both sexes or none and by those who did not know in whom they were interested than among those interested exclusively in the opposite sex. Among girls in upper secondary education, gender harassment and unwelcome sexual attention were reported less commonly by those interested solely in the opposite sex than by all other groups, and sexual coercion was reported less commonly by those interested solely in the opposite sex and those who did not know in whom they were interested than the other groups (Table 3). Thus, the first hypothesis was supported.

**Table 3.** Repeated experiences of gender harassment, unwelcome sexual attention and sexual coercion according to romantic (8th and 9th grade pupils) and erotic (upper secondary education students) interest among Finnish adolescents. (% (*n*/*N*)).

Has had a crush on or been in love with … /Is sexually interested in …	Opposite sex	Same sex	Both sexes	None	Doesn’t know	*p*
BOYS 8th and 9th grade						
Gender harassment	2.8 (498/18020)	26.5 (103/389)	21.8 (96/440)	2.4 (69/2899)	2.4 (58/2463)	<0.001
Unwelcome sexual attention	2.8 (517/18244)	27.0 (110/407)	22.1 (100/453)	2.8 (81/2934)	2.5 (64/2515)	<0.001
Sexual coercion	1.9 (353/18244)	25.1 (102/407)	19.2 (87/453)	2.0 (58/2934)	2.0 (50/2515)	<0.001
GIRLS 8th and 9th grade						
Gender harassment	2.8 (526/18764)	9.6 (35/364)	8.9 (142/1596)	1.9 (43/2235)	1.7 (32/1916)	<0.001
Unwelcome sexual attention	3.9 (735/18879)	8.7 (32/369)	10.0 (160/1602)	2.3 (51/2265)	1.7 (33/1933)	<0.001
Sexual coercion	1.4 (260/18879)	6.5 (24/369)	3.5 (56/1602)	1.2 (26/2265)	0.9 (18/1933)	<0.001
BOYS upper secondary						
Gender harassment	1.8 (561/30731)	16.5 (72/437)	13.2 (92/697)	13.1 (34/260)	13.4 (68/507)	<0.001
Unwelcome sexual attention	2.3 (704/31045)	18.0 (80/444)	14.6 (103/707)	17.7 (47/265)	14.1 (73/517)	<0.001
Sexual coercion	1.5 (472/31045)	13.5 (60/444)	11.3 (80/707)	14.7 (39/265)	12.2 (63/517)	<0.001
GIRLS upper secondary						
Gender harassment	2.1 674/31906	5.9 43/727	6.8 170/2490	5.2 18/343	3.0 31/1036	<0.001
Unwelcome sexual attention	3.6 (1146/32054)	4.9 (37/731)	9.5 (237/2500)	5.8 (20/345)	47 (49/1041)	<0.001
Sexual coercion	1.3 (409/32054)	2.2 (16/731)	3.3 (82/2500)	3.2 (11/345)	1.1 (11/1041)	<0.001

### Sex and age differences (Hypotheses 2 and 3)

Among boys in comprehensive school Odds Ratios for reporting different forms of sexual harassment were 10–17 –fold among those reportedly interested in the same sex and both sexes compared to those reportedly interested in the opposite sex, when only age was controlled for. Among comprehensive school girls, 2.4–4.6 -fold increased Odds Ratios for reporting the different forms of sexual harassment were seen among those with a romantic interest in the same sex in the age-adjusted models (Table 4).

**Table 4.** Odds Ratios (95% confidence intervals) for having repeatedly experienced gender harassment, unwelcome sexual attention and sexual coercion, according to romantic and erotic interest among 8th and 9th grade pupils of the 9-year comprehensive school in Finland.

Has had a crush on or been in love with …	Opposite sex	Same sex	Both	None	Doesn’t know
BOYS					
Gender harassment	ref ref ref	**13.2 (10.3–17.1)** **5.6 (4.0–7.8)** **5.3 (3.7–7.7)**	**10.3 (7.9–13.2)** **4.3 (3.1–6.0)** **4.0 (2.8–5.6)**	0.9 (0.7–1.2) 1.1 (0.8–1.5) 1.1 (0.8–1.5)	0.8 (0.6.1.1) 0.9 (0.7–1.3) 0.8 (0.6–1.2)
Unwelcome sexual attention	ref ref ref	**12.8 (10.0–16.4)** **5.8 (4.2–8.1)** **5.3 (3.7–7.8)**	**9.4 (7.3–12.1)** **3.9 (2.8–5.4)** **3.4 (2.3–4.9)**	1.0 (0.7–1.2) 1.2 (0.9–1.5) 1.1 (0.8–1.5)	0.9 (0.7–1.2) 1.1 (0.8–1.4) 1.0 (0.7–1.4)
Sexual coercion	ref ref ref	**17.3 (13.3–22.3)** **7.8 (5.4–11.1)** **7.5 (5.0–11.1)**	**12.3 (9.4–16.1)** **5.1 (3.5–7.3)** **4.2 (2.8–6.3)**	1.0 (0.7–1.3) 1.3 (0.9–1.7) 1.2 (0.8–1.7)	1.1 (0.8–1.4) 1.3 (0.9–1.8) 1.2 (0.8–17)
GIRLS					
Gender harassment	ref ref ref	**3.5 (2.4–5.2)** 1.9 (1.2–3.0)^c^ 1.8 (1.1–3.0)	**3.3 (2.7–4.1)** **2.0 (1.6–2.5)** **1.9 (1.5–2.4)**	0.8 (0.5–1.0) 0.9 (0.6–1.2) 0.9 (0.6–1.2)	0.6 (0.4–0.9) 0.6 (0.4–0.9) 0.6 (0.4–0.9)
Unwelcome sexual attention	ref ref ref	**2.4 (1.7–3.6)** 1.3 (0.8–2.0) 1.2 (0.7–1.9)	**2.7 (2.2–3.2)** **1.6 (1.3–2.0)** **1.6 (1.2–2.0)**	0.6 (0.4–0.8)^a^ 0.6 (0.4–0.9)^b^ 0.6 (0.4–0.8)^b^	**0.4 (0.3–0.7)** **0.4 (0.3–0.7)** **0.4 (0.3–0.6)**
Sexual coercion	ref ref ref	**4.6 (3.0–7.3)** 2.3 (1.3–4.0)^c^ 2.3 (1.3–4.2)	**2.6 (1.9–3.5)** 1.5 (1.1–2.1) 1.4 (0.9–2.0)	0.9 (0.6–1.3) 0.9 (0.6–1.4) 0.9 (0.6–1.4)	0.7 (0.5–1.2) 0.6 (0.4–1.1) 0.6 (0.4–1.1)

Note: Model (a) controlling for age; (b) controlling for age, depression, anxiety, delinquency; and (c) controlling for age, depression, anxiety, delinquency, family structure, mother’s education, father’s education and parental unemployment.

Bolded Odds Ratios are statistically significant at level *p* 0.001. ^a^*p* = 0.001; ^b^*p* = 0.004; ^c^*p* = 0.003.

Among upper secondary education boys, Odds Ratios for reporting experiences of sexual harassment were about 7–11-fold in all other sexual interest groups compared to exclusively heterosexual boys when only age was controlled for. Among girls in this older age group, Odds Ratios for reporting gender harassment were about three-fold among those reportedly interested in the same sex, in both sexes, and in none compared to those exclusively heterosexually interested. Odds Ratios for unwelcome sexual attention and sexual coercion were increased among those interested in both sexes (Table 5).

**Table 5.** Odds Ratios (95% confidence intervals) for having repeatedly experienced gender harassment, unwelcome sexual attention and sexual coercion, according to romantic and erotic interest among 2nd year students in upper secondary/vocational school in Finland.

Is sexually interested in …	Opposite sex	Same sex	Both	None	Doesn’t know
BOYS					
Gender harassment	ref ref ref	**10.7 (8.2–13.9)** **6.3 (4.6–8.7)** **6.0 (4.2–8.4)**	**8.3 (6.5–10.4)** **3.7 (2.8–5.0)** **3.4 (2.5–4.6)**	**8.2 (5.7–11.9)** **3.6 (2.2–5.8)** **3.7 (2.3–6.0)**	**8.4 (6.4–11.0)** **4.0 (2.6–5.7)** **3.3 (2.3–4.9)**
Unwelcome sexual attention	ref ref ref	**9.5 (7.4–12.2)** **6.1 (4.5–8.5)** **5.6 (3.9–7.9)**	**7.4 (5.9–9.2)** **4.0 (3.0–5.3)** **3.7 (2.7–5.0)**	**9.3 (6.8–12.9)** **5.5 (3.6–8.3)** **5.8 (3.7–8.9)**	**7.1 (5.5–9.2)** **3.6 (2.5–5.1)** **3.1 (2.1–4.6)**
Sexual coercion	ref ref ref	**10.2 (7.6–13.6)** **6.2 (4.3–9.0)** **5.5 (3.6–8.2)**	**8.3 (6.5–10.7)** **3.9 (2.8–5.4)** **3.6 (2.5–5.1)**	**11.3 (8.0–16.1)** **6.0 (3.7–9.6)** **6.2 (3.8–10.1)**	**9.1 (8.7–12.0)** **4.2 (2.9–4.3)** **3.4 (2.2–5.3)**
GIRLS					
Gender harassment	ref ref ref	**2.9 (2.1–4.0) 2.0 (1.4–2.8)** 1.9 (1.3–2.7)^a^	**3.4 (2.8–4.0) 2.2 (1.8–2.7)2.2 (1.8–2.6)**	**2.6 (1.6–4.1)** 1.6 (1.0–2.7) 1.5 (0.9–2.6)	1.4 (1.0–2.1) (0.7–1.6) 1.0 (0.7–1.6)
Unwelcome sexual attention	ref ref ref	1.4 (1.0–2.0) (0.7–1.4) 0.9 (0.6–1.3)	**2.8 (2.4–3.3)** **1.9 (1.6–2.2)** **1.9 (1.6–2.2)**	1.6 (1.0–2.6) (0.6–1.7) 1.0 (0.6–1.7)	1.3 (1.0–1.8) (0.7–1.4) 1.0 (0.7–1.4)
Sexual coercion	ref ref ref	1.7 (1.0–2.8) (0.6–1.9) 1.0 (0.6–1.9)	**2.6 (2.0–3.3)** **1.9 (1.4–2.4)** **1.8 (1.4–2.3)**	2.5 (1.3–4.5)^a^ 1.6 (0.8–3.2) 1.4 (0.7–3.0)	0.8 (0.5–1.5) 0.7 (0.4–1.3) 0.7 (0.4–1.4)

Note: Model (a) controlling for age; (b) controlling for age, depression, anxiety, delinquency; and (c) controlling for age, depression, anxiety, delinquency, family structure, mother’s education, father’s education and parental unemployment.

Bolded Odds Ratios are statistically significant at level *p* 0.001. ^a^*p* = 0.004

Thus, the associations between other than exclusively heterosexual romantic/erotic interests and experiences of sexual harassment were stronger among boys than among girls in both the younger (comprehensive school) age group and the older (upper secondary education) age group. Hypothesis 2 was supported. The associations between other than exclusively heterosexual interests and experiences of sexual harassment were not systematically and essentially stronger in the younger age group than in the older. However, as the questionnaires had been age adjusted and therefore romantic interests were elicited from the comprehensive school sample and erotic interests from the upper secondary education sample, conclusions based on age group comparison require caution. Hypothesis 3 was not supported.

### The role of confounding by emotional and behavioral disorders and socio-demographics (Hypothesis 4)

Among boys in comprehensive school the Odds Ratios seen in age-adjusted models were decidedly diminished when mental health variables and finally also socio-demographics were controlled for, but experiences of sexual harassment were nevertheless reported 3.5–7.5-fold more commonly by boys with romantic interests in the same sex and in both sexes. Among comprehensive school girls, the associations between same sex-interest and experiences of sexual harassment were leveled out when confounding was controlled for but being interested in both sexes also persisted as associated with gender harassment and unwelcome sexual attention in the final models (Table 4). In addition, girls reporting no romantic interest in either boys or girls and not knowing if they had had a crush on or been in love with someone displayed decreased Odds Ratios for unwelcome sexual attention. Among upper secondary education boys, even if controlling for confounding by mental health variables and socio-demographics weakened the first detected associations, other groups than those exclusively heterosexually interested had about 3–6-fold increased Odds Ratios for reporting various experiences of sexual harassment (Table 5). Among girls in this older age group, only those interested in both sexes had increased Odds Ratios for reporting different experiences of sexual harassment in the final models (Table 5). Hypothesis 4 was supported.

## Discussion

In a nationally representative school survey data, romantic and erotic interests in the same sex and in both sexes were in bivariate analyses associated with increased experiences of various types of sexual harassment (gender harassment, unwelcome sexual attention and sexual coercion) among adolescents in two age groups and educational stages, among both boys and girls. This concurs with earlier reports associating sexual minority status with subjection to sexual harassment in adolescence in samples collected from a few schools or through interest groups the United States and Canada (Gruber & Fineran, 2008; Mitchell et al., 2014; Williams et al., 2003). The associations detected were clearly stronger among boys than among girls. However, contrary to our hypothesis the associations detected between interest in same sex/in both sexes and experiences of sexual harassment were not essentially and systematically weaker in the older age group than in the younger, neither among boys nor among girls.

### Sex differences

Among boys in the younger age group the associations first detected between romantic interest in the same sex or both sexes and experiences of sexual harassment persisted when internalizing and externalizing symptoms and finally socio-demographics were controlled for, even if controlling for these confounders clearly weakened the associations. Among boys in the older age group the associations first detected between any other than exclusively heterosexual erotic interest and experiences of sexual harassment likewise persisted, although weakened, after the same confounders were controlled for. Among girls most associations first seen between romantic/erotic interest in the same sex or both sexes were leveled out when confounders were controlled for. Earlier studies on sexual harassment by sexual orientation have not accounted for confounding by mental disorders and socio-demographics and have thus not been able to confirm that the associations detected are independent main effects.

The sex differences in associations between sexual minority status and experiences of harassment may be due different attitudes towards male and female homosexuality and gender-nonconforming behaviors among males and females. Such attitudes may also result in greater minority stress among not exclusively heterosexual boys than girls.

Boys interested in the same sex or both sexes may express themselves in ways that make them susceptible to harassing behaviors. Research has suggested that behavior deviating from culturally accepted masculine norms in boys is less readily tolerated than deviating from the expected feminine behavior in girls (Ristori & Steensma, 2016). Self-expression not conforming to masculine norms may trigger aggression that serves to maintain heteronormativity (Chesir-Teran & Hughes, 2009; Poteat & Rivers, 2010). Sexual harassment has been seen as a mechanism for reproducing beliefs and attitudes that maintain rigid and unequal gender roles, even when targeted at men (Pina et al., 2009; Street et al., 2007). Self-expression of not exclusively heterosexually interested boys may challenge those rigid gender roles and therefore give rise to approaches that attempt to re-establish the norm.

Secondly, among girls, intensive dyadic friendships are common and developmentally normative (Miething et al., 2016; Selfhout, Branje, & Meeus, 2009; Shulman, Laursen, Kalman, & Karpovsky, 1997). Intensive friendships may even bear a close resemblance to romantic relationships (Diamond, 2002). Perhaps less attention is paid to romantic feelings between (dyads of) girls, and thus those feelings may not particularly challenge heteronormativity. In the present study romantic interest only was specifically elicited in the younger age group. Some of the adolescents reporting romantic interest in the same sex in early to middle adolescence may not actually be developing towards homosexual identity. If this is the case, young girls experiencing romantic feelings towards other girls may not have a minority identity and not experience minority stress. In the older age group, girls reporting sexual interest in both sexes stood out as more likely to have experienced gender harassment and unwelcome sexual attention, even when confounders were controlled for. In this age group, the focus was clearly on sexual interest, and it is, further, more likely that adolescents aged 16–18 also express their interest on a behavioral level, seeking intimate contacts. In this phase, bisexual girls may pose a greater challenge to the heterosexual norm, rendering them more likely to experience sexual harassment.

Of course, as our data is cross-sectional we cannot draw conclusions on causality. Due to the cultural phenomena that may render sexual minority boys more susceptible than sexual minority girls to actually being subjected to sexual harassment, boys becoming aware of their not exclusively heterosexual interests may also be more prone to experience minority stress (Meyer, 1995, 2013) than corresponding girls, be more vigilant of disrespect and discrimination, and report more experiences of sexual harassment, while exclusively heterosexual boys perhaps do not notice or nor perceive similar approaches as harassing. Girls sexually interested in both females and males may also differ from other girls in having more identity struggles and difficulties in being included, and this could make them more sensitive to sexual cues, and consequently remember and report more episodes of sexual harassment. Such sensitivity and vigilance could be understood in light of minority stress theory and internalized homophobia (Meyer, 1995, 2013; Szymanski et al., 2008), which has been suggested particularly strong among bisexual individuals (Kashubeck-West, Szymanski, & Meyer, 2008), who may perceive themselves as a minority not only in relation to the heterosexual peers but also to homosexual community.

### The role of age/developmental level

Contrary to our hypothesis the associations detected between interest in same sex/in both sexes and experiences of sexual harassment were not essentially and systematically weaker in the older age group than in the younger, neither among boys nor among girls. Given that from early to late adolescence, both cognitive abilities including perspective taking, emotional and behavioral controls and sexuality mature (Cacciatore et al., 2018 (under review); Moshman, 2011; Steinberg, 2005) it could have been expected that in peer groups of middle to late adolescents, sexual harassment behavior would decrease and the adolescents would be more careful not to directly or indirectly infringe the boundaries of their potentially more vulnerable peers, such as those belonging to sexual minorities. As to school bullying, such favorable development has been suggested (Kurki-Kangas et al., 2018). The same aspects of progressing development could also make sexual minority adolescents themselves more able to cope with minority stress and therefore less vigilant for heterosexist communications. However, this was not the case. This may suggest that heterosexism is still so deep rooted in contemporary Western culture that merely the progression of adolescents development that improves both behavioral controls and coping skills is not enough to reduce sexual minority youth’s experiences of sexual harassment.

### No or uncertain sexual interests

Among the younger age group in comprehensive school, aged on average 15 years, those not (yet) romantically interested in either sex and those not knowing if they had had a crush on or been in love with someone did not differ from those exclusively heterosexually interested as regards reporting experiences of sexual harassment. It seems plausible to assume that they have not yet entered the arenas of romantic encounters, are not noticed in a sexual way and are perhaps also insensitive to sexual clues. Among the older group in secondary education, aged about 17, however, boys reporting sexual interest in neither sex and those not knowing to whom they were attracted were as likely to report experiences of sexual harassment as those non-heterosexually interested. By age 17 the majority of boys in Finland have had intimate sexual experiences (Kaltiala-Heino et al., 2015; Savioja et al., 2015). The association between reporting no sexual interest or uncertainty about orientation and experiences of sexual harassment in the older age group could be explained in several ways. Those boys may, like their peers interested in the same sex, express themselves in ways that make them more susceptible to harassment. Sexuality as a developmental domain may be more stressful for them, and thus they may also be more sensitive to sexually loaded innuendos and perceive harassment more easily than actively interested (exclusively heterosexual) boys. It is also possible that they have been traumatized by harassing experiences and that this has blocked their sexual interests. Williams et al. (Williams et al., 2003) have likewise observed that adolescents uncertain of their sexual orientation reported bullying, sexual harassment, and dating violence more commonly than peers with a heterosexual orientation, and as commonly as young people identifying as gay, lesbian or bisexual. However, our analysis adds the finding that the meaning of not knowing or not being interested is different for adolescents of different ages, as is appropriate given that sexual maturation is an important facet of adolescent development, progressing from earlier to later developmental phases (Cacciatore et al., 2018 (under review); Savioja et al., 2015).

Interest in neither sex or uncertainly about one’s orientation were not similarly associated with increased experiences of sexual harassment among the older age group girls. Girls who are not interested sexually or are uncertain about their sexual interests perhaps do not challenge norms as do similarly oriented boys, because girls are still expected to be less sexually active than boys and are socially rewarded for chastity (Bordini & Sperb, 2013). For the same reasons, it may also be less stressful for girls themselves to not (yet) feel interested sexually even towards late adolescence, and thus they perhaps do not become sensitive to sexual innuendos as perhaps do boys in similar situations.

### The role of confounding

The confounding factors studied, particularly the emotional and behavioral disorders, explained a considerable share of the associations between sexual orientation and experiences of sexual harassment. Sexual minority status is per se associated with emotional and behavioral disorders in adolescence (Beaver et al., 2016; Marshal et al., 2011), as is subjection to sexual harassment (Kaltiala-Heino et al., 2016a). Controlling for confounding by emotional and behavioral disorders is a novel contribution of our study in research on sexual harassment among adolescents. Our results demonstrate that sexual harassment is a problem related to sexual minority status more markedly among boys, among whom the differences between crude and adjusted Odds Ratios were also greater, suggesting a stronger role of confounding in the disorders studied. Additional changes related to controlling for socio-demographics were modest.

### Implications for health psychology interventions

Subjection to sexual harassment is more common than being a victim of child sexual abuse or physical abuse (American Association of University Women, 2001; Kaltiala-Heino et al., 2016b; Stoltenborgh, van Ijzendoorn, Euser, & Bakermans-Kranenburg, 2011). Subjection to sexual harassment is stressful and associated with a number of negative behavioral and emotional consequences, such as fear and avoidant behavior in relation to school, emotional symptoms such as depression, anxiety and eating disorders, and behavioral symptoms such as conduct problems and substance use (American Association of University Women, 2001; Bucchianeri et al., 2014; Buchanan et al., 2013; Chiodo et al., 2009; Felix & McMahon, 2006; Goldstein, Malanchuk, Davis-Kean, & Eccles, 2007; Gruber & Fineran, 2007; Kaltiala-Heino et al., 2016a; Marshall, Faaborg-Andersen, Tilton-Weaver, & Stattin, 2013; Petersen & Hyde, 2013). Tackling sexual harassment thus likely offers approaches for promoting adolescent mental health and preventing the onset of disorders. On the other hand, it is also important to recognize those groups of adolescents at greatest risk of becoming targets of sexual harassment in order to offer adequate support and promote coping and self-protecting skills. Our findings suggest that young people belonging to sexual minorities, especially boys, constitute a particularly vulnerable group in this context. Health psychology interventions should focus on reducing sexual harassment experiences in sexual minority youth at macro (societal), meso (enviromental) and micro (client, patient) levels. At the societal level, sensitivity to heterosexism is needed (Kashubeck-West et al., 2008). Health psychology can make an impact by educating the general public and policy-makers about the negative impact of heterosexism and sexual harassment. To tackle the problem of sexual harassment at the community (meso) level, addressing the school atmosphere and promoting the inclusion of sexual minority youth is needed (Berger, Poteat, & Dantas, 2017; Kashubeck-West et al., 2008). For example, teachers and other school personnel do not always recognize homophobic language commonly used by adolescents (McCabe, Dragowski, & Rubinson, 2013). Counseling school personnel to increase their sensitivity to heterosexism and sexually harassing interactions can help to change the level of bias and reduce harassing interactions. Establishing activities that reduce prejudice and promote sexual equality in schools, for example Gay-Straight alliances, can both reduce sexually harassing behaviors towards sexual minority youth and offer sexual minority youth empowering experiences and help them to cope effectively (Kashubeck-West et al., 2008; Wormington, Anderson, Schneider, Tomlinson, & Brown, 2016). Both actual changes in the psychological environment and better coping likely reduce minority stress. At micro level, health psychology interventions can make an impact by encouraging sexual minority youth to engage in activism and work to change the heterosexist environment, by helping sexual minority youth to cope more effectively and defend their boundaries (Dunn & Szymanski, 2018). Deconstructing internalized heterosexism (homophobia) should also be a focus in individual counseling (Kashubeck-West et al., 2008). Deconstructing internalized homophobia will likely reduce minority stress and increase well-being by both increasing self-acceptance and relieving excessive vigilance and expectations of discrimination. Further, given the strong role of emotional and behavioral disorders in the association between sexual orientation and sexual harassment experiences, interventions should also focus on recognizing and treating mental health problems in sexual minority youth and older adolescent boys confused about or unaware of their sexuality.

### Methodological considerations

A strength of our study is its uniquely large and nationally representative population data comprising adolescents in two different age groups and developmental phases.

In this data reporting experiences of sexual harassment was less common than has previously been reported, and when focusing on experiencing the various forms of sexual harassment ‘repeatedly’, differences between boys and girls were small and even such that boys reported harassment more frequently. Many studies have focused on any experiences in a given time frame (American Association of University Women, 2001; Bucchianeri et al., 2014; Chiodo et al., 2009; Petersen & Hyde, 2009) and therefore focusing on experiences that have occurred ‘repeatedly’ obviously results in smaller numbers. However, gender harassment and unwelcome sexual attention were in this sample less common than in an earlier Finnish report using data from an earlier School Health Promotion sample even when response categories ‘sometimes’ and ‘repeatedly’ are summarized (Kaltiala-Heino et al., 2016b). Two methodological issues may affect this. In the present data, questions related to sexual harassment were placed in a section focusing on delinquency and crime, whereas earlier they were placed among questions on sexual health. It has been shown that the context in which experiences of sexual harassment are elicited influences how they are reported. The ‘framing’ – the name of the survey, the contents of the rest of the survey, and the associations created by knowing who are responsible for the survey may all affect how respondents understand given questions and how they respond (Galesic & Tourangeau, 2007). Secondly, the sexual harassment questions now had response options no, sometimes and repeatedly, whereas the previously offered alternatives were no and yes. This may impair comparability. However, from 2013 to 2015, a reduction in reported experiences of sexual harassment was seen when comparing exactly the same questions (https://www.thl.fi/fi/tutkimus-ja-asiantuntijatyo/vaestotutkimukset/kouluterveyskysely/tulokset/tulokset-aiheittain/tapaturmat-ja-vakivalta), which suggests that the phenomenon may also really be decreasing in Finland.

Sexual orientation has been defined as comprising aspects of attraction (to whom one is attracted), behavior (with whom one has sexual encounters) and identification (what group one considers oneself to belong to) (Marshal et al., 2011). In different studies, these aspects have been used in different ways. In the present study attraction only was in focus. A more comprehensive understanding of sexual harassment and sexual orientation could be obtained if all aspects of sexual orientation were elicited.

It has been shown that in survey studies adolescents rather exaggerate than underreport their belonging to minorities. In the present study we attempted to control for invalid!! responding by using as a screen a combination of responses that are unlikely to hold true all at the same time, as suggested by Robinson-Cimpian (2014). Indeed, this screen caught a disproportionate share of adolescents reporting non-heterosexual interests and experiences of sexual harassment. The use of such a screen can be seen as a strength of the present study.

Sexual harassment is a concept close to or overlapping with both child sexual abuse and bullying, of which a considerable share is sexual in nature (Ashbaug & Cornell, 2008; Stoltenborgh et al., 2011). Child sexual abuse and school bullying have both likewise been associated with negative mental health outcomes (Kaltiala-Heino & Frojd, 2011; Wilson, 2010) and are reported more commonly by sexual minority youth (Friedman et al., 2011; Katz-Wise & Hyde, 2012). Research related to these topics can further increase the understanding of sexual harassment among sexual minority youth.

## Conclusion

Sexual orientation has stronger associations with experiences of sexual harassment among adolescent boys than among girls. Compared to peers reporting exclusively heterosexual romantic and erotic interests, adolescent boys interested in the same sex or both sexes report excessive experiences of sexual harassment. Among girls, such associations are weaker. Also, not being interested in either sex or not knowing to whom one is attracted are associated with excessive experiences of sexual harassment in older boys, but not in younger boys. Boys interested in the same sex and both sexes and older boys uncertain of their preferences or not sexually interested may be perceived as more threatening to the heterosexual norm than corresponding girls. Health psychology interventions need to aim at reducing heterosexism and promoting the inclusion of sexual minority youth in order to reduce sexual harassment targeted at them. In working with sexual minority youth, deconstructing internalized heterosexism is important, and given the mediating role of emotional and behavioral disorders between sexual minority status and experiences of sexual harassment, it is important to recognize and address mental disorders in sexual minority youth.
